# Yield Estimation and Visualization Solution for Precision Agriculture

**DOI:** 10.3390/s21196657

**Published:** 2021-10-07

**Authors:** Youssef Osman, Reed Dennis, Khalid Elgazzar

**Affiliations:** Faculty of Engineering and Applied Science, Ontario Tech University, Oshawa, ON L1H 7K4, Canada

**Keywords:** precision agriculture, computer vision, deep learning, data analysis, geospatial mapping

## Abstract

We present an end-to-end smart harvesting solution for precision agriculture. Our proposed pipeline begins with yield estimation that is done through the use of object detection and tracking to count fruit within a video. We use and train You Only Look Once model (YOLO) on video clips of apples, oranges and pumpkins. The bounding boxes obtained through objection detection are used as an input to our selected tracking model, DeepSORT. The original version of DeepSORT is unusable with fruit data, as the appearance feature extractor only works with people. We implement ResNet as DeepSORT’s new feature extractor, which is lightweight, accurate and generically works on different fruits. Our yield estimation module shows accuracy between 91–95% on real footage of apple trees. Our modification successfully works for counting oranges and pumpkins, with an accuracy of 79% and 93.9% with no need for training. Our framework additionally includes a visualization of the yield. This is done through the incorporation of geospatial data. We also propose a mechanism to annotate a set of frames with a respective GPS coordinate. During counting, the count within the set of frames and the matching GPS coordinate are recorded, which we then visualize on a map. We leverage this information to propose an optimal container placement solution. Our proposed solution involves minimizing the number of containers to place across the field before harvest, based on a set of constraints. This acts as a decision support system for the farmer to make efficient plans for logistics, such as labor, equipment and gathering paths before harvest. Our work serves as a blueprint for future agriculture decision support systems that can aid in many other aspects of farming.

## 1. Introduction

Precision agriculture is defined as the integration of technology in different facets of agriculture. Many aspects of agriculture benefit significantly from automation; from monitoring soil and crop conditions, to assisting in irrigation, plant treatment and harvest. One of the earliest successful works in precision agriculture is the use of wireless sensing to monitor soil conditions. For example, Corwin et al. [1] found that the electrical conductivity of the soil highly affects its productivity through measuring it via soil sensors. There are other works that utilize sensing for detecting abnormalities within the crop. Tian et al. [2] aimed to focus on weed detection as it is a common problem with any field of crops. They introduce a sensor capable of detecting weed fields, so that once a farmer sees areas that are affected by weed infestations, they can more swiftly and precisely treat it before it spreads to other patches. In general, understanding conditions of the crop and soil aids the farmer in making informed decisions, directly affecting farming efficiency and profitability. In this paper, we specifically target crop yield estimation, and the use of spatial data to visualize the acquired yield.

Crop yield estimation, which we also refer to as fruit counting in our work, is the process of producing an estimated count of fruit or vegetables within their respective field. The farmer can then use the count to decide on what to do for harvest. Specifically, resources such as man power, trucks and other vehicles, storage and packaging. For our paper, we develop a fully DL-based framework to perform yield estimation and support harvest decision making. We divide our framework into two main components: yield estimation and yield mapping. We perform yield estimation by counting fruit yield through videos. That way, our solution is robust enough to work on any type of fruit from video feeds as opposed to limited static images. This is quite efficient and scalable with the integration of mobile sensors (e.g., robots and drones) that would autonomously navigate through crop fields and capture video footage. We then incorporate spatial information about the yield to recommend optimal placement of harvest containers to reduce the number of required bushels and save their collection efforts, enabling farmers to make better use of their resources while optimizing the harvesting process.

## 2. Related Works

Crop yield estimation relies on using image processing algorithms detect the crop from imagery and count the number of detections to give the estimate. In earlier works, detections are made by distinguishing between the crop’s textures and background textures. Wang et al. [3] use visual cues to segment apples from images, specifically the hue, saturation and intensity to detect both red and green apples under specific lighting. Pothen et al. [4] present a keypoint detection algorithm that detects potential fruit regions based on intensity profile then uses high-dimensional features in those detected regions to classify them as fruit or not fruit. Roy et al. [5] estimate apple count from images as well as their diameters by segmenting apple clusters in an input image using a nonlinear optimization method. Then feed the segmented images into a Structure from Motion (SfM) pipeline to reconstruct them up to scale. The aforementioned techniques do not produce state-of-the-art results, are very specific to the selected crop (e.g., apples), and cannot be easily translated into yield estimation for other types of crop without a drastic change in the algorithm. Additionally, these algorithms can perform poorly outside of controlled environments, such as light intensity. In our work, the proposed models can be used with any fruit, given the appropriate dataset is provided to train the model in different circumstances, such as poor lighting conditions and occlusion behind another fruit or leaves.

Deep learning is on the rise in computer vision, with numerous state-of-the-art object detection and image segmentation algorithms utilizing deep learning models showing promising accuracy. Furthermore, one of the main advantages of deep learning is generalization, if the model works on one type of object, then it’s quite likely to work on other objects. It only needs to be retrained with a suitable dataset relevant to the domain of application. Rahnemoonfar et al. [6] prove this by performing fruit detection on seven different fruits using the Faster R-CNN object detector which is trained on the respective fruit. Bargoti et al. [7] use Multi-Layered Perceptrons and Convolutional Neural Networks (CNNs) to perform image segmentation to detect and count fruits from images. Sa et al. [8] perform tomato counting using a CNN model that’s a modification of Inception-ResNet [9]. Notably, the data they used for training is simulated by generating synthetic images of tomatoes, producing favorable test accuracy on real images of tomatoes. Chen et al. [10] present a deep learning pipeline for counting apples and oranges. An image of the crop is input into a blob detection neural network that is a fully convolutional network [11] which outputs a segmented image to distinguish fruit pixels from non-fruit pixels, noting that there could be a cluster of fruits that’s referred to as a blob. They then use a count neural network, which is another CNN that takes bounding boxes around each detection, and estimates the count of fruit in that box. These works produce great accuracy with good performance and potential for generalization, showing that deep learning is a viable methodology for yield estimation. However, they all work on images, not videos, of crops. This is an important issue to discuss because, as footage of the crop is typically taken in video form. In our work, we use the detection model within a tracking pipeline, which allows the system to keep track of detections across video frames and count new detections only once.

Liu et al. [12] address counting through videos by using a fully convolutional neural network (FCN) for object detection, and optical flow using Kanade-Lucas-Tomasi (KLT) tracker. However, their tests on apples were run under controlled illumination. Moreover, KLT-based optical flow tracking has shortcomings: it relies on the brightness of different regions in the frames remaining consistent, and that the object motion in that region has to be consistent. Any change of lighting or sudden change of motion will cause the tracker to suffer. Their work also included the use of SfM to avoid counting apples in different tree rows, which works well but the algorithm itself is very computational intensive. In our approach, we use a single-shot-detector (SSD) which is considered the state of the art solution to object detection problems. Additionally, we developed a custom tracking pipeline that leverages deep learning and intersection over union (IoU) matching in order to properly track objects through sudden motions, and changes in appearances. We also design a light-weight solution to resolve apples in back rows being unintentionally detected. Our dataset and tests are run on apple trees in natural illumination during daytime.

## 3. Materials and Methods

**Figure 1:**
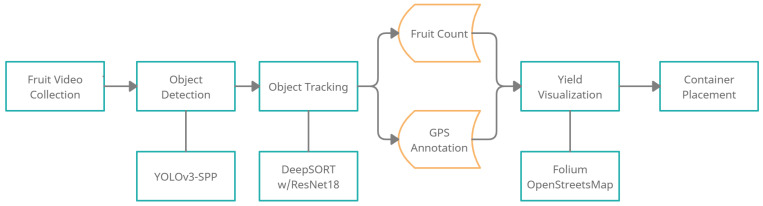
An end-to-end overview of the proposed smart harvesting pipeline.

We provide a high-level view of our pipeline in Figure 1. We begin by inserting video frames into our system. Object detection is run on each frame in order to detect the fruits within the frame, however, as discussed earlier, detecting the fruits is not enough to count the fruits in a video. The detections are passed onto our modified tracking model. The tracker is responsible for creating associations between the frames. For example, if fruit “a” is present in the first and second frames, then the tracker associates between them and gives them an ID of 1. Once an individual fruit is identified and tracked across the frames, it is counted, and a total yield estimate is provided at the end, however during the counting process we also perform GPS annotation. Following the GPS frequency, the count within a time period and its respective set of frames (e.g.,: every one second), is recorded alongside the current GPS point. We create a file with GPS points and counts at those points which we then use to visualize on a map, so the yield and its location are presented in a user-friendly manner to the farmer. We then add the function to optimally place containers used for harvest, such as apple bushels, on the map to complete our smart agriculture solution. In this section, we explain each step in our fully automated approach to yield estimation and visualization.

### 3.1. Fruit Detection

We start our pipeline by detecting fruits frame-by-frame. This is primarily an object detection task, where the desired objects (in our case, the fruits we are counting) are detected using bounding boxes. The expected output is the pixel-wise coordinates of a bounding box that surrounds each fruit, completed with a classification of what is inside the box and the confidence score of the detection. Due to the prevalence of existing powerful general use object detection models, we explore the possible options and make a decision based on accuracy, performance, robustness and ease of integration with our tracker.

#### 3.1.1. Model Selection

Convolutional neural network (CNN)-based object detectors have been able to achieve state-of-the-art performance on various benchmark datasets. As discussed in Section 2, the basic structure of a CNN object detector contains two parts: the backbone [13,14,15,16,17,18] which is responsible for extracting features from the image(s), and the head [19,20,21,22] which is responsible to predict bounding box locations and class values. Recently, CNNs have been fit with intermediate blocks between the backbone and the head; commonly called the neck [23,24,25,26,27,28] which is responsible for collecting feature maps from multiple stages of feature extraction or feature refinement for feature layers. Commonly, the goal of the intermediate neck layer/blocks is to improve accuracy whilst minimizing the incurred overhead. In Section 2, we explored various object detectors, each with its own unique architecture and set of advantages and disadvantages. With single stage detection (SSD) models showing high accuracy without processing speed trade-offs, we discard multi-stage detection models as they show significantly slower performances in comparison. We compile reported performance of the best object detector candidates in Table 1 and analyze their performance in terms of Average Precision (AP) and Framerate Per Second (FPS).

We chose to use You Only Look Once version 3 (YOLOv3) with spatial pyramid pooling (YOLOv3-SPP) [19,29] as the back-end object detector for our pipeline. Table 1 shows that YOLOv3-SPP has the best trade-off for accuracy and speed compared to other current state-of-the-art object detectors. For example, EfficientDet-D3 shows the highest AP of 47.2%, however the FPS performance is notably lower than both YOLOv3 models. We observe that RetinaNet boasts the highest FPS with AP that rivals YOLOv3, however the results are somewhat skewed by the use of NVIDIA TensorRT (TRT). With the use of TensorRTthe network’s inference time could potentially decrease without affecting the accuracy. However, we do not use TensorRT in our work because it is still highly experimental. The framework is very hardware demanding as it only works on certain NVIDIA GPUs and is only compatible with limited CUDA versions. More importantly, YOLOv3 is a heavily studied object detector that is used in many research and industry applications- many bugs and potential issues are ironed out in comparison to fresher models.

#### 3.1.2. YOLO3 Model Architecture

**Figure 2:**
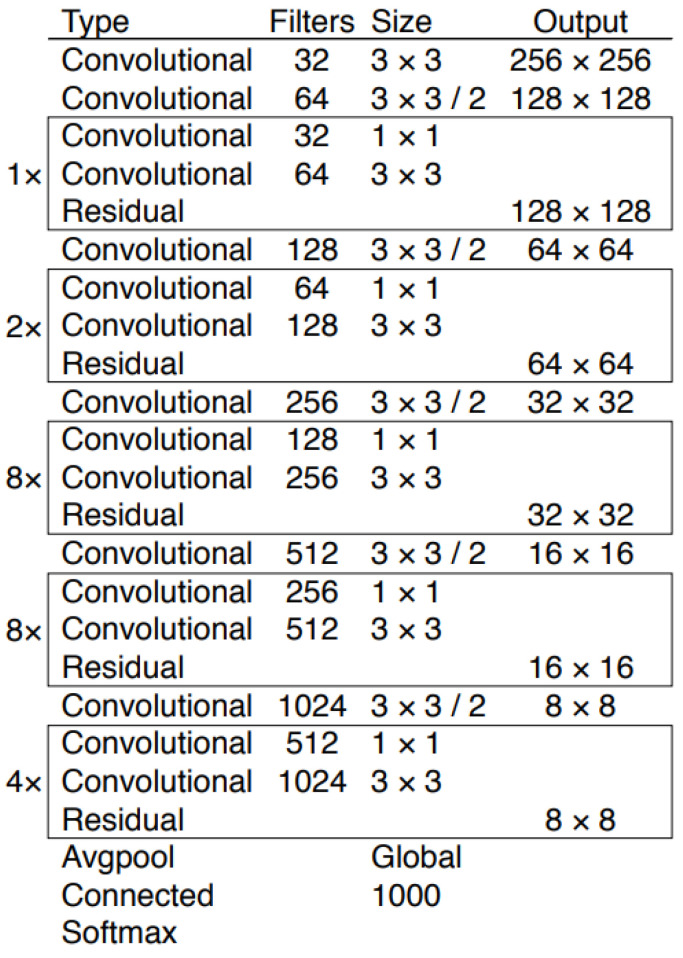
Darknet53 architecture that is used as YOLOv3’s backbone [19].

The YOLO object detector is divided into two components: feature extractor (the backbone) and detector (the head). The feature extractor is responsible for getting a feature map of the image. YOLO uses a CNN model called DarkNet53 that uses 53 layers as shown in Figure 2. The architecture is fairly straightforward with multiple convolutional layers to process the input, and residual layers, which are also known as skip layers, in-between. Firstly, convolutional layers use filters to analyze blocks of an input matrix, starting with the initial input image. Filters are considered the weights within the context of CNNs. They are small two-dimension matrices that are multiplied with each block of the input matrix. After all blocks of the input are used in the computations, the output is a new matrix that contains feature information. This process can be repeated multiple times by stacking subsequent convolutional layers. The output of the convolutional layer can be considered a mathematical representation of the image that’s used to classify the object within the image. Theoretically, the deeper the network is, the more accurate the classification will be. However, that’s not always the case, hence the introduction of residual layers [31] which are also used in Darknet53. In traditional neural networks, where each layer feeds directly into the next layer, residual networks allow for some layers to be skipped. This combats the vanishing gradients problem, where changes in the weight parameter (gradients) become so small that they disappear and that information becomes lost. Thus, during back-propagation no useful information will be sent back, crippling the training process. With skip connections, these small gradients can back-propagate through shorter paths, allowing for less changes and that information becomes preserved. The outputs of convolutional layers are considered the feature maps, which are used in the next stage.

**Figure 3:**
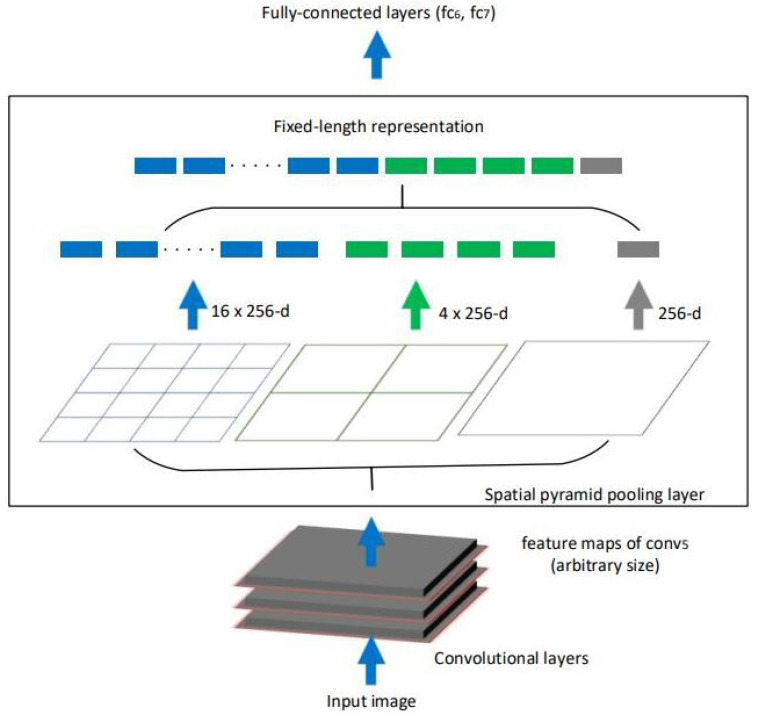
Visualization of SPP, reproduced from [23].

Detectors in the YOLOv3 leverage the output of each of the last 3 convolution stages. Three stages are selected to allow for multi-scale detection, where smaller objects are easier to detect in earlier stages, and bigger objects are easier to detect in later stages. In the YOLO model we have integrated, the spatial pyramid pooling (SPP) approach is used. SPP is based on the SPM model [32]. It focuses on improving classification and detection on a multi-scale level. SPP utilizes the output feature maps of different stages within the DarkNet CNN (specifically, the outputs sized 32 × 32, 16 × 16 and 8 × 8). After the feature maps are extracted, max pool operation is performed. The outputs are then concatenated together to form a long single vector similar to Figure 3. By using multi-scale features, the model is able to gather spatial information that significantly improves scene interpretation and detection of objects in various aspect ratios and scales. In practical settings, different fruits have different sizes (e.g., apples are smaller than pumpkins), plus the perspective of the footage can affect the fruit size as well (e.g., aerial views of pumpkins lead to pumpkins appearing smaller in size). As such, it is vital that our detector has the versatility to function accurately with any object size. Additionally, the size of the vector is fixed length, combined with the fixed scales of the design, it allows the model to work on images of any size.

In YOLO, the input image is divided into a 2-dimensional grid containing *N* × *N* cells (*N* being the grid size). Each cell within the grid is analyzed simultaneously through the feature extraction and detection explained above. Following the anchor box detection method, anchor boxes are assigned to each cell in the output matrix of the convolution stages. These anchor boxes have different aspect ratios and sizes. During the learning phase, the anchor box with the best intersection with the ground truth bounding box is considered the correct prediction. Thus the information predicted for each box is the location of the box (pixel wise center coordinates of the box denoted by *x* and *y*), whether the box contains an object or not, and what class this object belongs to.

#### 3.1.3. Correcting Detections

**Figure 4:**
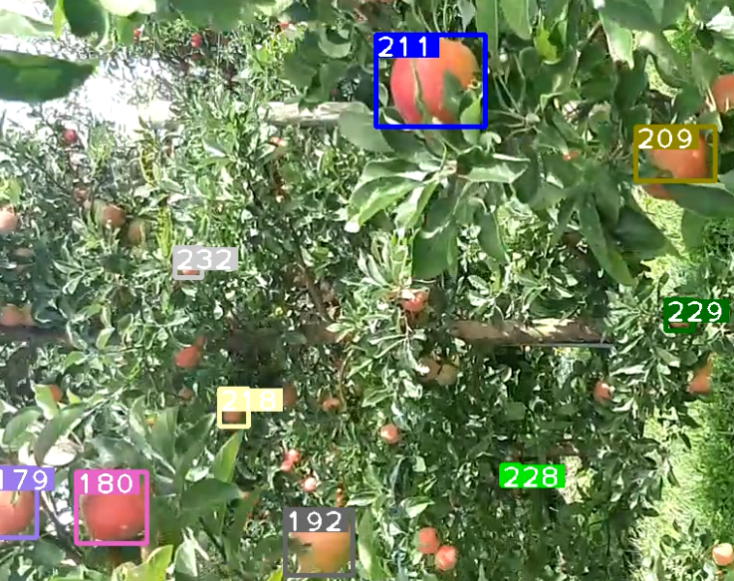
From the apple orchard video used, some apples from a back row tree are being detected and tracked.

In our implementation with apple data, we noticed that apples from other tree rows are being detected which can be seen in Figure 4. This is not intended as apple orchards are divided into rows of trees, and we perform the counting task on one row at a time. To solve this problem, we developed a lightweight thresholding mechanism to correct the detections returned by YOLO. We tested the detection model standalone and analyzed the average bounding box sizes of the distant apples specifically, and set bounding box height and width thresholds accordingly. Any bounding box that has a height and width below the threshold (30 pixels) is discarded before moving onto the next step.

For our work, we use YOLOv3 to detect apples, oranges and pumpkins. We obtain the x, y coordinates of the center of the box; the width and height of the box; the class it belongs to; and the class confidence score. All this information is then used for tracking. We opted not to change the architecture of the backbone as the use of default configurations allow us to improve the accuracy of detection directly through the use of transfer learning, where we can simply finetune the original weights pretrained on the MSCOCO dataset, which already contains plentiful fruit data, using much smaller datasets.

### 3.2. Fruit Tracking

#### 3.2.1. Tracker Selection

Because of the speed-accuracy trade-offs of tracking-by-detection methods, we decided to choose a two-stage tracking method opposed to a single, or unified detection and tracking method. However, the specific tracking method is not an integral component of this research, any robust tracking method could be interchanged with our chosen tracking method.

Tracking-by-detection, or two-stage tracking methods (Sometimes also called Multi-Hypothesis-Tracking (MHT) [33]) integrate a tracking framework with a detection framework. The detection framework produces bounding boxes, class scores, and confidence scores which are then fed into the tracking framework to make associations between objects detected in the current frame and objects detected in previous frames, in order to update tracklets and apply re-identification.

This is often done by modelling the problem as a data association problem between tracklets and detection’s. SORT [34], DeepSORT [35], IOU/V-IOU [36,37] are three popular online methods that are commonly used for two-stage tracking frameworks. Of course, two-stage methods rely quite heavily on the quality and consistency of their inputs. Low quality detectors can cause fragmentation of the tracks and inconsistent detection’s can result in ID switches or lost tracklets. A stable frame rate is also important, where smaller steps (or higher frame rate) allows for more accurate associations between well defined tracks.

Simple online and realtime tracking (SORT) [34] was a pivotal breakthrough for research into tracking-by-detection approaches. This “simple” approach first detected objects using a generic object detection pipeline, namely Faster R-CNN [38]. Then, in order to generate the associations between detection’s and tracklets, SORT used the detected bounding boxes geometry by predicting the bounding boxes future locations using a Kalman Filter [34,39], which iteratively refined said predictions throughout successive frames. The cost matrix, used to assign detected bounding boxes with pre-existing tracklets, was computed using the IoU between each detection and all predicted bounding boxes, where a minimum IoU threshold was imposed to reject poorly defined assignments. The assignment was then solved using the Hungarian Algorithm [34].

The IOU tracker [36] is another popular online tracking-by-detection method. Under the assumption of a robust detector that successively produces “high overlap” detection’s, the IOU tracker then tracks these detection’s in a similar fashion to SORT [34] by associating detection’s with the highest IoU to existing tracklets. As was the approach in SORT, the Hungarian algorithm was used to solve said assignments. Note that no image information is used by the IOU tracker, nor does the IOU tracker attempt to predict the future locations of past detection’s as is done in SORT.

In a similar fashion to SORT, one of the biggest drawbacks of the IOU tracker was missing detection’s, which would lead to significant fragmentation and high ID switches. The V-IOU tracker extended the IOU tracker by incorporating a visual single-object tracker [37] in an attempt to mitigate these issues. The philosophy is relatively straight-forward. If no detection is available for association with an existing tracklet, then continue said tracklet using a visual tracker, rather than relying on the [object] detectors inputs.

The IOU/V-IOU trackers are able to generate association’s and track objects at incredible speeds, limited only by the computational requirements of the detector. However, they do not work well within domains where high-occlusions are commonplace. Moreover, because of the assumption that both IOU/V-IOU trackers make with respect to the “high overlap” of objects, they are not well suited for applications where large camera movements are frequent.

As such, in this work we extract the bounding boxes from the detector and perform a tracking function to assign unique id’s to the objects across various frames. The algorithm we use for MHT is a modified version of DeepSORT [35], as DeepSORT provides a great trade-off between accuracy and real-time tracking speeds whilst also dealing well with occlusions.

#### 3.2.2. DeepSORT

DeepSORT is an extension of the Simple Online Realtime Tracking (SORT) [34] algorithm. It relies on creating ’tracks’ which represent the tracked objects and applying Kalman Filter to predict the next states of objects. DeepSORT then associates between the same objects in different frames by using distance metrics, one is based on the motion of the object and the other is based on the appearance of the object. The model gives favorable performance only using the appearance as a metric [35], so for our work, we only use the features produced by the CNN which represent the appearance of the object.

Following the original SORT, DeepSORT performs Kalman Filtering and defines the tracking scenario on an 8-dimensional state space (*x*, *y*, *a*, *h*, ẍ, ÿ, ä, ḧ), which respectively denote the center *x* and *y* coordinates of the bounding box, the aspect ratio and height, and their velocities. A linear Kalman Filter following a constant velocity model is used, which is a standard for object tracking [40]. The bounding box coordinates (x, y, a, h) which are received from YOLO are used without modification. DeepSORT then uses deep learning and CNN features to create associations between new detections and the current tracked objects by using a minimum cost algorithm to obtain the objects with the minimum distance with their associated tracks. In the following sections, we describe the components of the tracker in detail.

#### 3.2.3. Track Initialization

After we obtain the first bounding boxes from YOLO, the first step in the DeepSORT pipeline is initializing the tracked object for the detections. The track is given a number of attributes including:

- Track ID as an identifier;
- Age which denotes how many frames this track existed in, initialized as 1;
- Number of hits which is incremented every time this track is successfully associated, initialized as 1;
- Feature matrix which is the output of the CNN and represents the object is appearance;
- Track state which represents the current state of the track so it can be confirmed or deleted, initialized as tentative;
- Mean and covariance matrices which are used for the Kalman Filter that are updated with every prediction.

#### 3.2.4. Convolutional Neural Network

DeepSORT’s main feature is the use of deep convolutional features. When an image is input into a CNN, the features of the image are analyzed within each layer and then an activation function such as Softmax is applied to the output vector to classify the object once all the convolutional layers have been completed. Intuitively, this means that the vector output after convolutional layers describes the appearance of whatever is in the image. This was leveraged in YOLO in order to detect objects and it is also used in DeepSORT’s pipeline as the appearance metric for tracking. The use of appearance allows for better tracking in case of a random object motion and occlusion, which are both very common challenges in tracking applications.

The original model used is a wide residual network that is been trained on a person re-identification dataset [41] which is limited to people tracking (specifically, pedestrians) making it unsuitable for any fruit data. Thus, in this work we insert a different CNN model that shows a state-of-the-art performance called ResNet18 [31]. We chose to use ResNet rather than creating a CNN model from scratch for several reasons. First, ResNet has been utilized in a wide range of applications for years and its performance and limitations have been well investigated. Second, ResNet comes with a variety of setups, primarily varying depths. This allows us to choose a depth based on our requirements. We chose ResNet18 for our system because it has the best performance and fruit characteristics aren’t complicated enough to justify more layers. We also performed some initial tests and found no difference in counting accuracy between ResNet18 and ResNet101. Fourth and most importantly, ResNet provides weights that are pretrained on the ImageNet classification dataset [42], a massive dataset with 1000 classes, including fruits such as apples for our application. This eliminates the requirement to train the DeepSORT feature extractor and allows the DeepSORT pipeline to be used for practically any fruit without modifying the tracking model and saving time and resources that would otherwise be spent on training.

Table 2 shows the architecture of ResNet18. There are 5 convolutional layers, and a skip connection is in between every layer. After the final convolutional layer, there is an average pooling layer which leads to an output vector of size [1, 1, 512] that is input into a fully connected layer for classification. Since we do not perform classification and we need the appearance descriptor of the object, we take the output of the average pooling layer as our final output to be treated as the feature map of the object.

#### 3.2.5. Association and Counting

The model associates between tracks and detections in a frame using their respective feature maps. This is an assignment problem to be solved using the Hungarian algorithm, which is a standard minimum cost algorithm. It is vital to use an efficient algorithm as the number of tracks and detections can increase substantially, where every track needs to be compared against every detection. The problem shown in Equation (1) is formulated as follows: given an array of tracks (*T*) of size t, and an array of detections (*D*) of size d, we compute a new cost matrix (*C*) where index [*i*, *j*] is the cosine similarity between the feature map of *T(i)* and the feature map of *D*(*j*). After the matrix is complete, we simply find the minimum cost for each track in *T* out of all the detections and that forms the association.

(1) C(i,j) = min(1−cos_similarity(D(j),T(i)))

The Hungarian algorithm can be divided into 4 main steps. We start off by providing our input, which is a 2-dimensional matrix sized *n* × *n*, each cell representing the cost between the track and the detection. The first step is to obtain the minimum value in each row and subtract it from each cell in its respective row. The second step is to do the same for each column, i.e., subtracting the minimum value in each column from all elements in that column. The third step is to mask rows and columns to cover all the zeros that were computed due to the previous two steps. Then there are two scenarios: (1) the number of lines required to cover zeroes is *n*, and in this case the optimal assignment is done and the algorithm ends, (2) otherwise, we search for the smallest uncovered index and subtract it from all uncovered indices. If the entire matrix is covered, then the algorithm ends, otherwise repeat this step until completion. The final optimal assignments are the cells with the value ’0’. Meaning, if C(1, 3) = 0, where 1 is the index of Track 1, and 3 is the index of Detection 3, then Detection 3 will be associated with Track 1.

**Figure 5:**
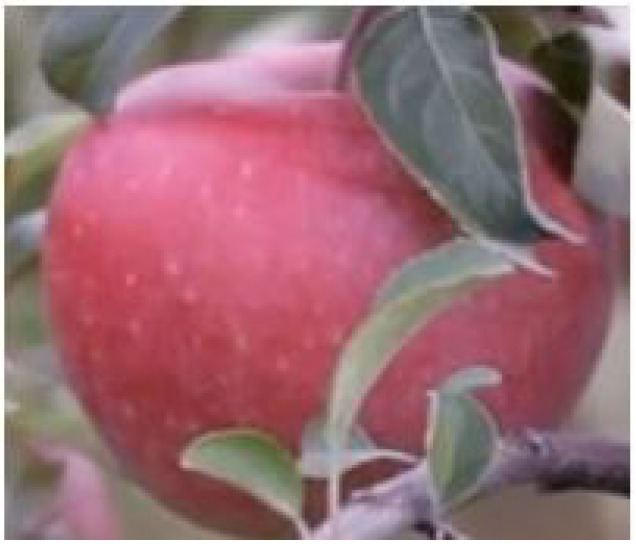
First apple detected in first frame and is inserted into the tracker which identifies it as track 1.

**Figure 6:**
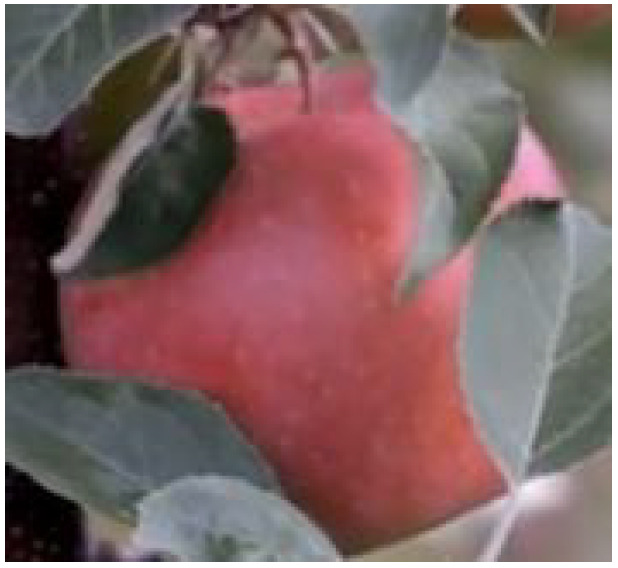
Apple identified in second the frame, noted as detection 1 is tested for association.

**Figure 7:**
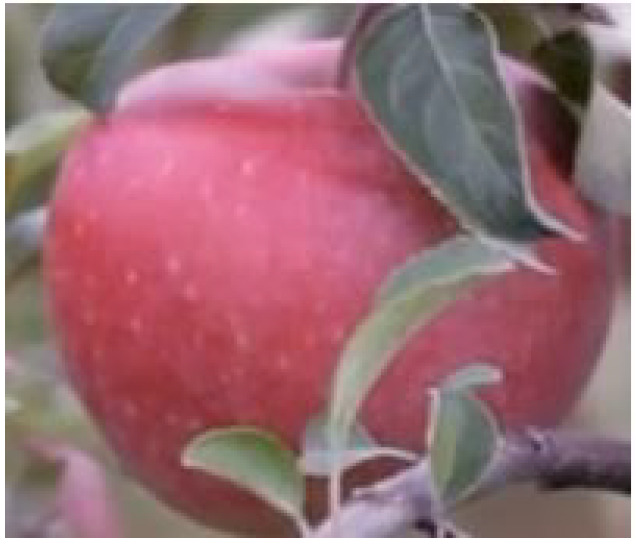
Second apple identified in the second frame, noted as detection 2 is tested for association.

The cost value in our context is the cosine distance between CNN features. After a feature map is extracted from the ResNet18 model we implemented, we obtain the cosine distance using Equation (2). The cosine distance, also known as cosine similarity, is a method to compute how similar two vectors are in an inner product space. Essentially, the cosine similarity measures the angle between two vectors and applies the cosine function to it. This determines where both vectors are pointing and whether they’re pointing in the same direction. This is applicable with CNNs as we can obtain feature maps in the form of a two-dimensional matrix from the convolution and pooling operations that we can then flatten into vectors and compare between them. For example, Figure 5 shows the saved image which we can assume as track 1. If there are two new detections added to our tracker, shown in Figure 6 and Figure 7, we now compute the cosine similarity according to Equaton (2). The result will look something like: Similarity(i, j) = [0.64, 0.99]. That means the similarity between Track 1, Detection 1 is 0.64, and the similarity between Track 1, Detection 2 is 0.99. Then, once a minimum cost algorithm is performed based on Equation (1), Track 1 will be associated with Detection 2, which is the correct assignment as they are quite clearly the same apple with only a slight shift in motion. This operation can be extended to any tracks and detections that will be added.

(2) $$\cos\left(t,e\right)=\frac{te}{∥t∥∥e∥}=\frac{\sum_{i=1}^{n}t_{i}e_{i}}{\sqrt{\sum_{i=1}^{n}{\left(t_{i}\right)}^{2}}\sqrt{\sum_{i=1}^{n}{\left(e_{i}\right)}^{2}}}$$

As the objects get analyzed and tracked throughout the frames, there are three states that could be assigned to each track in every frame:

1. Tentative: this is a temporary state. It means that a new detection is potentially a new object to be tracked. New detections remain tentative until they are either matched with an existing track or are turned into their own new track.
2. Confirmed: this means that the track is created and is confirmed as a new object to have entered the scene. The detection changes its state from *Tentative* to *Confirmed* and is ready to have new detections associated with it.
3. Deleted: this means that the track has left the scene and is no longer tracked. It is considered deleted and will no longer be considered during the matching stage.

Therefore, a track is not immediately confirmed upon association. The track needs to have a number of hits greater than 2 to change its state to *“Confirmed”*. This helps to avoid brief false detections. Secondly, not all tracks will be associated. The distance (where 0.0 denotes an exact match, and 1.0 denotes completely different features) needs to meet a certain threshold to be considered as a match. Following the original implementation, the maximum distance is 0.15, meaning that for anything higher, the match will be discarded. If a track is not associated, it will remain saved for a number of frames until its age surpasses the preset maximum age, which we set as 30. In a 30 FPS video, if an object is not associated for 1 s, it is discarded as it is considered to have left the scene. Before unmatched detections are initialized as new tracks, they undergo IoU matching first. Specifically, the IoU between two tracks bounding boxes is computed, and if the IoU is 0.3 or greater (meaning that there is some intersection), the tracks are associated with each other. This improves the tracking robustness because sometimes a fruit can be clear in one frame, obscured in another (thus, having different appearance features), then clear again in the following frame. The IoU tracking helps keeping track of that apple when an appearance association can briefly fail. Another important parameter in DeepSORT includes a minimum confidence, which is the minimum accepted confidence from the detector. Anything lower than the minimum confidence causes the detection not to be inserted into the tracking pipeline. We selected a score of 0.4, as fruits that were partially hidden by leaves or branches, or fruits that were blurred due to camera motion were indeed detected, but with notably lower confidence. Thus, a lower threshold allows for such apples to be considered. It was also observed that lower thresholds than 0.4 include wrong detections, mostly leaves that were mistakenly detected as another fruit and have very low confidence scores.

It is important to note that, while every new track has its own ID, we can’t use it as a count reference because some tracks are still tentative and will be deleted if they aren’t properly associated. As a result, apples are counted only when the track is confirmed.

### 3.3. Geospatial Mapping of Fruit Count

The combination of geospatial information with crop analysis is critical to smart harvesting as it provides farmers with rich information to optimize their resources and make informed decisions on how to plan for pre- and post-harvesting. GPS technology can enable feature maps in the field such as visualization of soil happiness, tree density, targeted treatment and fertilization, container placement and many more. In this work, we record GPS points while recording videos of the apple rows to support these features. GPS capturing is synchronized with video capturing so we are able to match the GPS coordinates with the video during the counting process.

During data gathering, the GPS device consistently records its current coordinate. After data capturing and analysis are complete, we annotate the counts from the video frames with their respective location on a map. To accomplish this, we record the frequency at which the GPS points are captured and assign a GPS point to a set of frames. In this research, we record 1 GPS point every 3 s (GPS period). Our video is recorded at 30 frames per second, which means 1 GPS point is assigned to every 90 frames (3 ∗ 30). We formulate an equation for the frames per GPS point in Equation (3). We then implement a counter alongside our fruit counting pipeline that increments until it reaches the maximum frames per GPS point. As such, the count is precisely recorded with the exact geolocation. The counter is reinitialized after each GPS annotation and the following GPS point is ready to be assigned.

(3) frames_per_GPS_point = GPS_period∗frames_per_second

**Figure 8:**
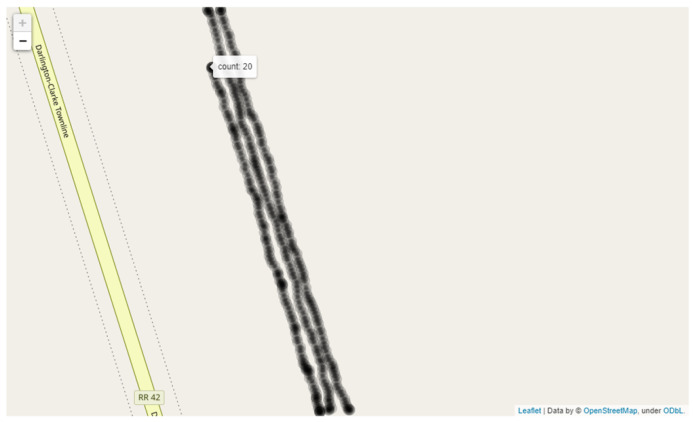
Mapping the GPS data after counting.

The GPS coordinates and their recorded count (shown in Table 3) are used to provide better visualization of the data. Our aim is to create a map containing the yield information at a point-by-point basis on a map. To do so, we use the Folium package in Python and create a map containing GPS points with a tooltip containing the count in an OpenStreetMap template. A runtime visualization is shown in Figure 8.

### 3.4. Container Placement Optimization

We utilize the geospatial information and yield estimation to provide farmers with a smart harvesting system that will assist them with harvesting logistics. To demonstrate this feature in this work, we perform container placement optimization where we provide a strategy to place a minimum amount of containers in optimal and fixed locations across the field when preparing for harvesting.

To formulate the objective function for this task, we use the sum of the vector representing the number of possible containers we can use Equation (4). To clarify, we have $y_{k}\in [0,\;1]$ for k = 1, 2, ..., K, where *K* is the upper-bound, or maximum number of containers to place. This means that if there’s 1 container assigned, it would be a summation of 1 + 0 + 0 + …, leading to a total of 1. If there are 2 containers assigned, then the summation would look like 1 + 1 + 0 + …, leading to a total of 2 and so on.

(4) $$\min\sum_{i=1}^{K}y_{k}\;$$

Our constraints following this are as follows: we have i = 1, ..., n apples, we introduce a variable $x_{ik}$ that is used to determine whether or not apple *i* is assigned to container *k*. Note that an apple must be assigned to only one container. To ensure that every apple is assigned to a container only once, we introduce the following constraints:

(5) $$\sum_{i=1}^{K}x_{ik}=1\;$$

What this constraint implies is that every apple “k” is assigned to its respective container “i”. Moreover, “*x*” is a matrix that contains information about each apple and gives apple “k” the value of 1 at its respective container i, and zero in the rest of the column. For example, in the below matrix, with 3 apples and 3 containers, apple 1 is assigned to container 1, apple 2 is assigned to container 3, and apple 3 is assigned to container 2.

$$\left(\begin{array}{ccc} 1 & 0 & 0\\ 0 & 0 & 1\\ 0 & 1 & 0\; \end{array}\right)$$

The last constraint that is needed is the distribution of weight. We check to see that the weight of the apples assigned in every container does not exceed the maximum weight a container can carry. This is done by taking the sum of the product of the total number of apples in a given container “*x*” with respect to the weight of each apple “*w*” and determining if it is less than or equal to the maximum weight “*m*” of a given container “*y*”. This is represented as follows:

(6) $$\sum_{i=1}^{n}w\cdot x_{ik}\leq m\cdot y_{k}\;$$

Finally, in our implementation, we monitor the distance between each two containers to ensure they don’t exceed a set maximum distance. Once the second container reaches the distance threshold, it is immediately placed even if it is not full. This is to ensure that large containers are not too spread from each other, leading to inconvenience for the pickers during harvesting.

The final output is the optimal number of required containers and their GPS coordinates on a map. The visualization is implemented using the Folium package in Python. By offering this service to farmers, they will be able to plan harvest routes, equipment and container expenses, and labor requirements more efficiently, thus transforming the future of precision agriculture with advanced technologies.

### 3.5. Dataset Preparation

The massive success of deep learning is generally attributed to its abilities to learn a wide variety of features from data. As such, preparing a thorough dataset is the most important step within a deep learning-based pipeline. In our work, we train the object detector model first on separate small fruit datasets without training the feature extractor in the tracking module. The reason for using small domain-specific custom datasets is that modern CNNs are generally pretrained heavily on massive image datasets, namely ImageNet for feature extraction [42] and COCO for object detection [40]. ImageNet is a massive dataset that consists of millions of annotated images and around 20,000 categories, from humans to various types of fruit. ResNet (our CNN feature extractor) is one of the models that has already been trained on ImageNet, and functions efficiently for fruits. This allows us to save time and effort needed to prepare a fruit dataset just for tracking and allows our tracking module to be used immediately for most fruits. On the other hand, the COCO dataset is geared towards object detection and segmentation tasks, containing hundreds of thousands of images with annotated objects and around 81 classes. Modern object detectors are pretrained on the COCO dataset, however, as it is relatively on a smaller scale, it is generally required to fine-tune the weights of object detection models to work on the desired objects. While we investigate the accuracy of using pretrained weights in a couple of cases (apples and oranges) that will be shown in the following section, we also prepare three small fruit datasets (2 apple and 1 pumpkin) to train our YOLO model with.

To begin with, we prepare two separate apple datasets using footage taken from an apple orchard containing around 150 & 100 images, respectively. The first was used for early experiments and was taken at a time when the apples were not fully ripe, and the second one was used for yield estimation of full rows of apples completely ripe. To annotate our images, we use a label software called YOLOLabel. To annotate images for model training, we draw boxes around the desired objects provide a label to it (i.e., the desired class). Each image has a respective text file that contains the annotation details. Specifically, the text file contains the center x and y coordinates of the box, the width and height dimensions, and the class ID. In our work, we load the apple images and specifically annotate the apples. Given that we run our work on apple videos, we configured the model so that we only have 1 class: *“apple”*, so all our objects have a class ID of 0. If we use the YOLO model with pretrained COCO weights, then *“apple”* ID is 48.

It is crucial to properly annotate images as such annotations determine what the network learns. The network is efficiently capable of learning simple features, where apples are obvious and not obscured. However, different image quality and context conditions in which apples might not be very clear to the object detector must be considered for efficient training. In apple orchards, it is quite common to find apples in contexts that affect their visual appearance. For traditional methods, poor lighting or occlusion would pose significant challenges for appearance-based detections, however, with deep learning we can train our network to learn such features. As such, we need to consider the following conditions while annotating the custom dataset: apples that are hidden by leaves, apples that overlap with each-other, apples under different lighting conditions, and other conditions that could potentially affect their visual appearance. Table 4 highlights the main different conditions that we’ve addressed during annotation. By labeling the apples within these different contexts, we significantly improve the model’s robustness whilst addressing key challenges (mainly occlusion and brightness) with fruit detection.

Since we aim to develop a general fruit detection approach, we also prepared a small pumpkin dataset that consists of 30 images of pumpkins. The images mostly consist of aerial views of pumpkin patches. Unfortunately, we couldn’t acquire more data as pumpkin data is severely limited. We use the same software package for annotation and follow the same labeling philosophy discussed above.

## 4. Results

### 4.1. Experiments on Apple Tree Segments

For evaluation, we compute the accuracy by comparing between the yield predicted by the model, and ground truth count which is performed manually by a human.

Table 5 shows the result of running our pipeline on apple videos containing smaller segments of the trees. We ran the experiments over 3 different model configurations: finetuned weights, pretrained weights, and pretrained weights corrected by our mechanism. We compute the L1 Loss between the predicted and ground truth, and compute the accuracy using the following equation where GT is the ground truth, and L is the computed loss.

$$\mathrm{Accuracy}=\frac{\mathrm{GT}-L}{\mathrm{GT}}\;*100$$

As expected, using just YOLO’s pretrained weights lead to a significant over count of 181 apples. This is due to how YOLO was trained on a massive dataset (MSCOCO) that featured apples of various sizes. Figure 4 shows a video frame where a distant apple tree has 3 apples that were detected and tracked. At first, we aimed to solve the problem by increasing the minimum confidence needed for the detection to be tracked. However, that didn’t help as there were other cases where the apple detection had lower confidence due to being occluded by other objects or, more commonly, blurred due to camera motion. Thus, the solution needed to tackle distant apples specifically. We ran the detection model on the same video and output all of the detections into the file, and we analyzed the detections against their respective apples to see the average size of a distant apple. Once we got an estimated size (30 pixels width and height) for the apple, we used that threshold in our correction mechanism.

Using YOLO’s pretrained weights with the correction mechanism yielded a substantially better result, where the model under-counted by 43 apples, giving an accuracy of 87.43%. We looked at how the detection was behaving, and we observed that while distant apples were no longer being detected, the detection model would sometimes struggle with occluded apples. The detector itself also yielded average confidence levels, which did not cause an issue in our experiment due to our minimum confidence being fairly low, but would perform worse if the minimum confidence is increased. To address these issues, we use transfer learning and fine tune YOLO’s weights using the dataset discussed previously. We make sure to include occluded apples and apples with different shading. We also changed the configuration of YOLO to only include the apple class instead of MSCOCO’s 80 classes. Our fine tuned weights showed an improvement, with the model under-counting by only 29 apples, with our accuracy being 91.5%.

### 4.2. Experiments on Full Rows of Apple Trees

**Figure 9:**
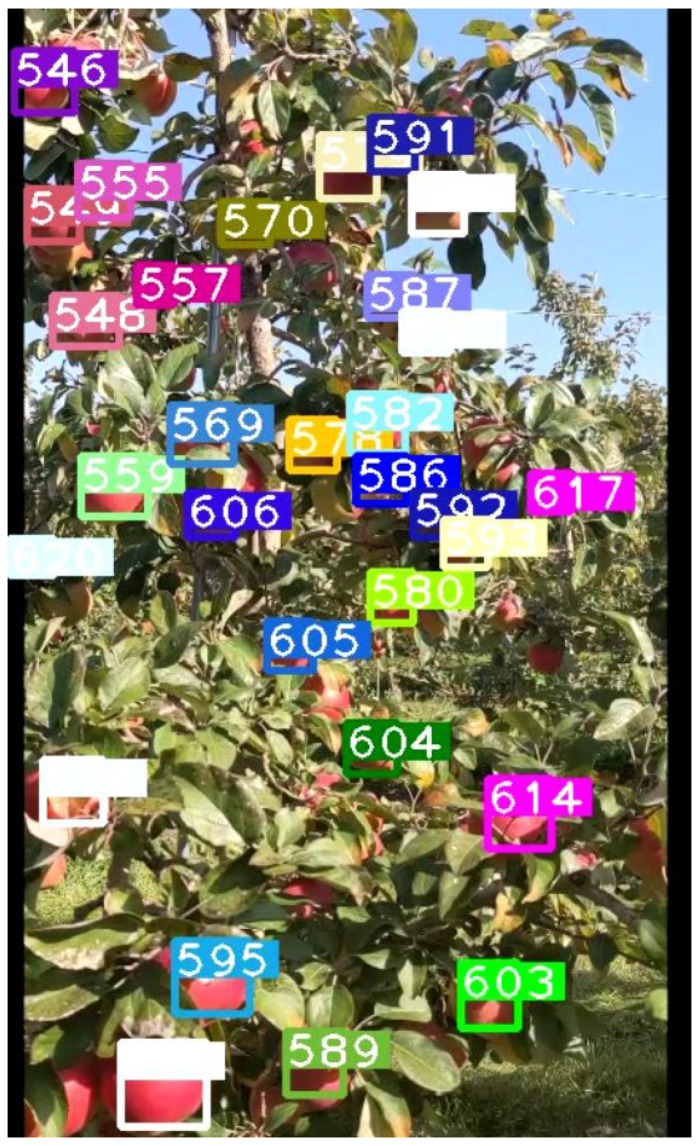
A frame taken from the video of the apple tree during runtime, just in this frame there are approximately 30 apples being tracked, in addition to the saved tracks that are not currently detected. There are several apples that are largely occluded for which one of the following scenarios could be true: (1) previously detected and counted before becoming obscured; (2) will be detected next with the camera motion or with a clearer angle; (3) will fail to be detected leading to a loss in counting accuracy.

**Figure 10:**
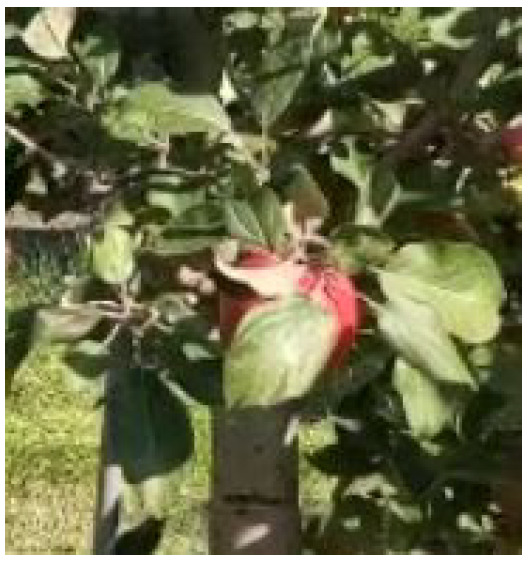
The leaf covers the apple and is predominantly visible. We avoid annotating such apples to avoid mistakenly detecting leaves as apples and will instead rely on the angle eventually making the apple clearer.

**Figure 11:**
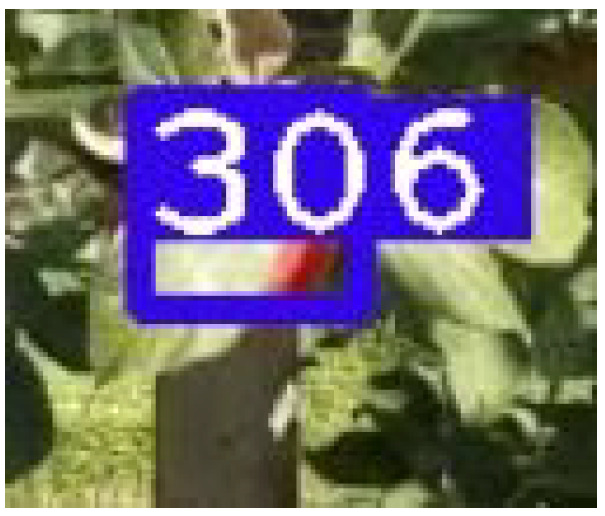
The apple does indeed become clearer in the following frame, allowing for detection to occur and the tracker to save and count the apple.

In our initial experiments on apple trees, we focus on small segments of the trees (with roughly 300 apples) to try and catch as many different cases of apple visibility as possible. We then expand the use of the framework with much larger and more realistic segments of apple trees (with roughly 3–4 k apples) to test the scalability and practicality of the framework. In this experiment, we perform counting on 3 separate full-length rows of apples, catching the whole trees. As our primary goal is to test for scalability, we train our model first with imagery of full apple trees and run our experiments using exclusively the fine tuned weights. Our findings are presented in Table 6. Despite each row has different lightnings due to the direction of sunlight and the fact that trees themselves have subtle differences in their shape and density, the result has a little variance ranging between 90.6% to 95.8% and the accuracy closely resembles our initial experimentation that yielded a 91.5% accuracy with fine tuned weights. We inspected each video analysis to understand the reason behind the difference in accuracies. We found that in apple row 1, there are a few trees that are extremely dense on leaves and their apples can barely be noticed, thus a large number of apples are completely undetected in comparison to other rows. For apple row 2, the sunlight was striking the camera directly, which makes all the apples appear darker than they are which presents a visual problem that affects the detector’s performance. For this row, we observed that our annotating approach and diversifying the dataset with different conditions as discussed in the previous section have improved the accuracy to 93%, otherwise it could have been much lower. Finally, apple row 3 had good lighting and most of the apples are clearly viewed within the trees, thus the framework achieved an accuracy of 95.8%. This shows that, despite the significantly larger amount of apples per frame, such as in Figure 9, the framework remains robust and efficient. Similarly, visual challenges, such as apples being occluded by leaves, can be observed in Figure 10. As the camera moves and more of the apple pixels get revealed, the apple is detected and appropriately tracked as seen in Figure 11. However, like our previous experiments, largely obscured apples pose a challenge to our framework due to our reliance on detectors to accurately pick up all visible apples. Depending on the density of the tree, as well as farming practice (i.e., whether the tree is pruned or not), this challenge can either lead to insignificant accuracy loss, or more noticeable under-counts. From this point on, more training and a larger dataset would lead to small increases in accuracy. However, we need to avoid overfitting and ensure that detectors can still pick up subtle differences between each different row in the orchard. Further, our modified DeepSORT algorithm enabled us to only retrain the selected object tracker (in our case, YOLOv3) and avoid retraining or modifying the tracker.

### 4.3. Other Fruit Counting: Oranges and Pumpkins

**Figure 12:**
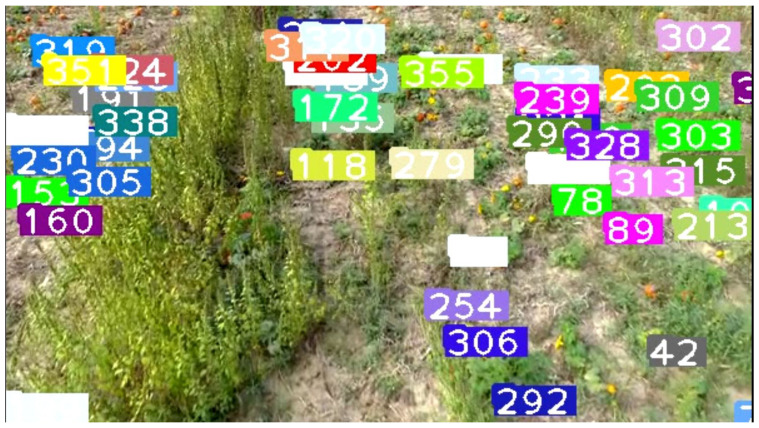
A frame taken from the video showing the view of the pumpkins and all of the current detections, the numbers denote the track ID.

**Figure 13:**
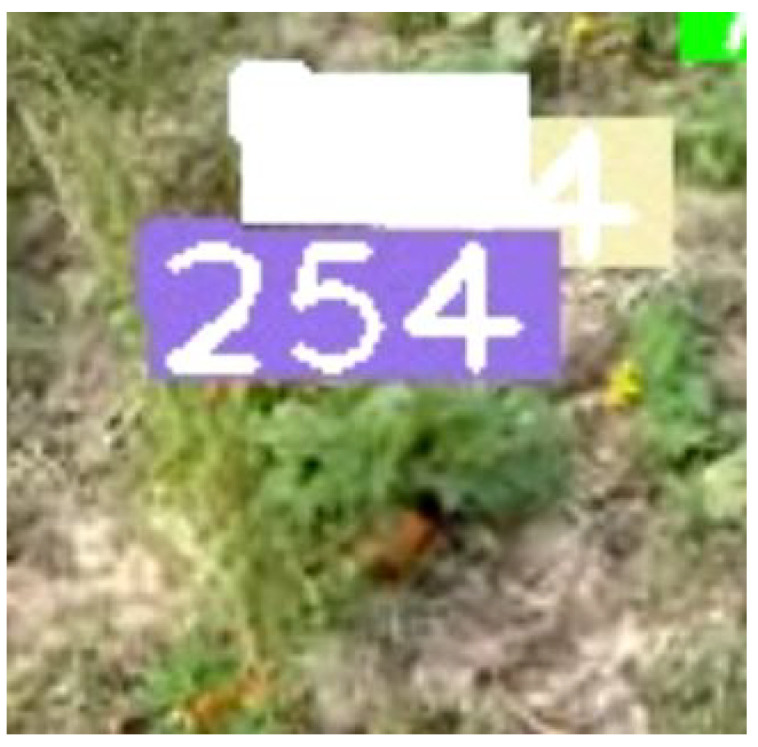
Another pumpkin that is hidden and is not currently detected nor counted.

**Figure 14:**
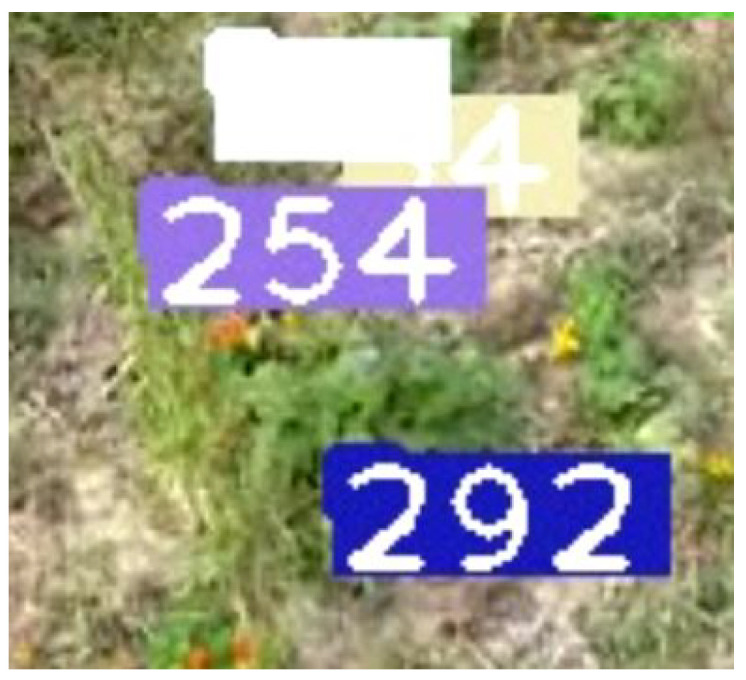
The change in view as the drone flies forward allows more of the pumpkin to be seen, thus is successfully detected and given a track ID.

**Figure 15:**
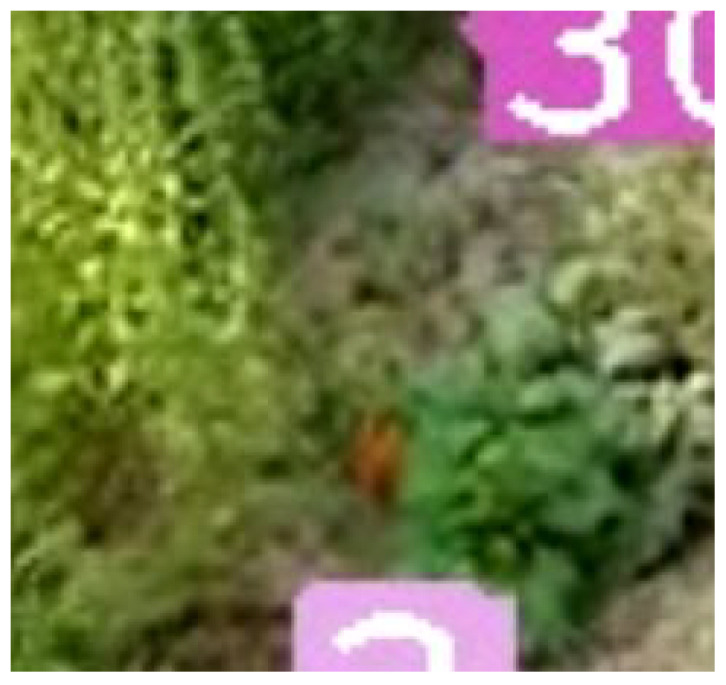
Pumpkin is mostly hidden and is hard to be seen due to little to no lighting, in further frames the pumpkin only becomes more hidden and is never detected.

**Figure 16:**
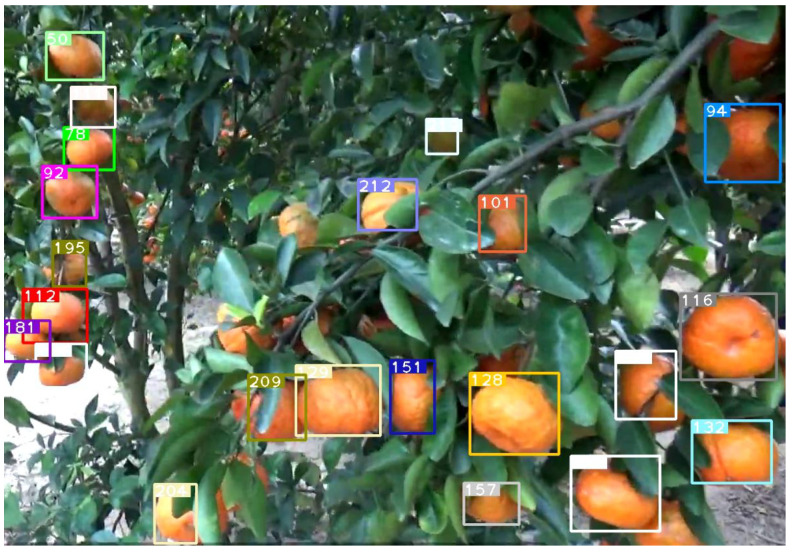
A view of detected oranges in the tree, numbers denote track ID.

**Figure 17:**
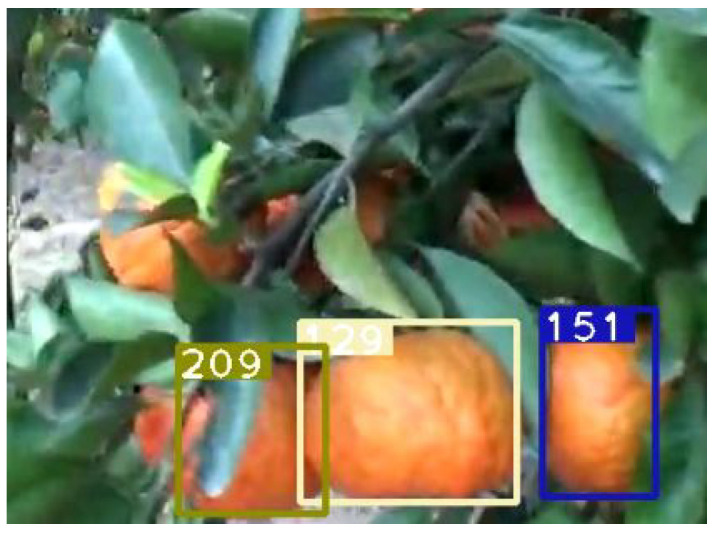
The majority of uncounted oranges are heavily obscured behind other oranges and leaves, the YOLO pretrained weights don’t fully accommodate brightness and occlusion challenges.

This section reports the experimental results and performance analysis of the proposed framework applied to other fruits (orange and pumpkin specifically) to prove its generalization and feasibility in precision agriculture. Table 7 presents the results of our experiments on videos of oranges and pumpkins. Pumpkin footage is captured by a drone, which provides another interesting perspective of applying the framework on far aerial views. Figure 12 shows a frame from the footage that displays the aerial view and current detections and tracks. It can be noticed that very few pumpkins are not yet tagged. There are two common scenarios that might occur in this use case: (1) pumpkins that are not visible in one frame will eventually become more visible in future frames and are appropriately detected and tagged. This is a very common scenario and is shown in Figure 13 and Figure 14. (2) There are few pumpkins that can hardly be seen and are never detected, thus are never tagged by the tracker, such as in Figure 15. This is similar to the cases in apple counting. During our pumpkin experiments we faced major issues in finding video data with pumpkins and used a relatively limited number of short videos and images to train our model. Despite this limitation, our modified DeepSORT tracker functions near-perfectly with the detections provided and achieves a high accuracy of 94.9%. Due to the formation of the pumpkin patch, it is a straightforward task to detect each individual pumpkin from the top-down view that the drone provides. Additionally, pumpkins have a distinct and relatively large shape and its bright orange color stands out from the surrounding background (grass and leaves). On the other hand, the base YOLO model already has an oranges class and the default pretrained weights are trained to recognize oranges. As such, we use the pretrained weights for the detector to show that the modified DeepSORT tracker can efficiently run on any object without further training or modification. However, the tracker works with oranges and produces a count, as seen in Figure 16, the accuracy is lagging a little bit, showing an accuracy of 79.3%. An analysis of the footage during runtime quickly shows oranges that are heavily occluded by other oranges or branches, as seen in Figure 17, failed to be detected even throughout the movement of the camera. This problem existed in our earlier analysis on apples using pretrained weights, however, our data augmentation and annotation approach explained in Section 4.2 tackle this challenge very well and lead to higher accuracy. Unfortunately, collection of real orange footage is challenging due to the limitations of orange orchards in our area and lack of such footage or available data online.

## 5. Container Placement Results

We apply our container placement algorithm on apple row 3 from our second experiment. While the feed of apple row 3 undergoes counting, the GPS coordinates are synchronized and annotated with the counts as discussed in Section 3.4. After the yield estimation process is complete, we process the geospatial and count information to begin proposing the number of required containers and their optimal placements. We apply different sets of constraints when it comes to the maximum weight of the container, and the maximum distance between each placement. We included containers that can occupy up to 300 and 1000 apples and set our maximum distance to 40 feet and 100 feet. In addition to checking the location and number of containers, we also check the utilization of a container. For example, if 1000 apples are assigned to a container of size 1000, this means the container is 100% utilized and we achieve the minimum number of containers by having as many fully utilized containers as possible. We show the container placement locations and container utilization in Table 8, Table 9 and Table 10. Note that the pair (300 apples and 100 feet) yielded the same result as when the distance was set to 40.

**Figure 18:**
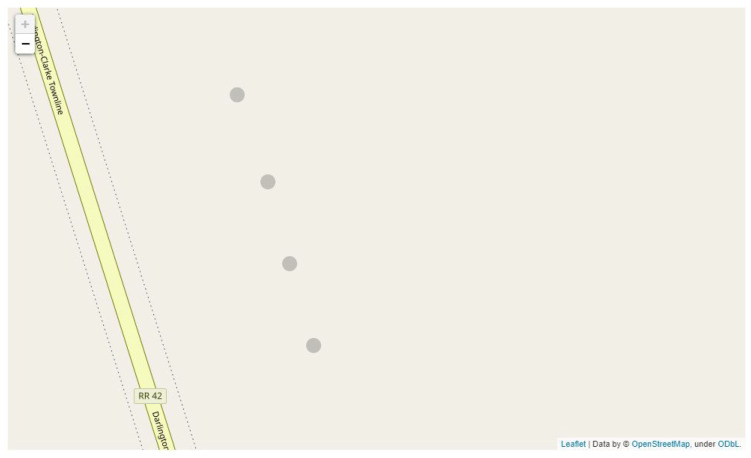
The container placements visualized using Folium and OpenStreetsMap template. The distance between the containers is evenly spaced across the row, ensuring harvesters will have a container near them.

**Figure 19:**
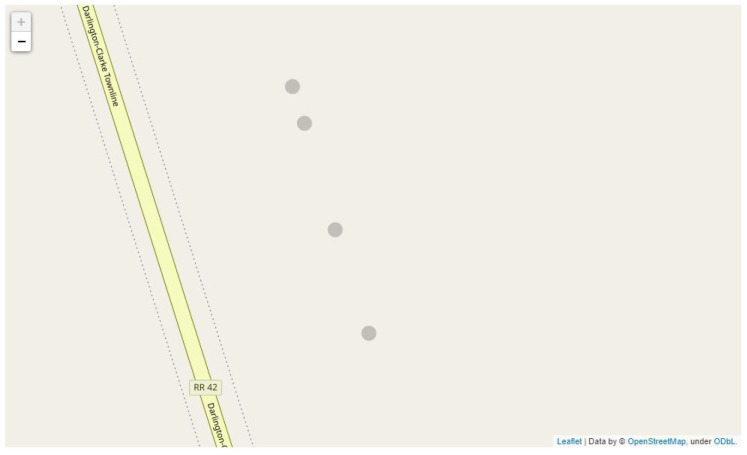
The distance between the containers is uneven, with the last two containers being close to one another. This means that harvesters between the 2nd and 3rd boxes will walk longer distances. There might also be a crowd around the 3rd and 4th boxes as they are fairly close to one another.

We can observe that in the case where the maximum distance was set to 100 feet, all of the containers were fully utilized relative to the apples being assigned regardless of the maximum capacity. However, there are some concerns that with larger sized containers (possibly even greater than 1000), there will be too much distance between the harvester and the container in which they collect the apples. When we set the maximum distance to 40 feet instead, we note a more uniform distribution of the apples across the containers as seen in Table 9. This also reflects on the mapping of the containers seen in Figure 18, where containers have more even spacing when the distance constraint goes into effect as opposed to Figure 19. We conducted additional testing with larger size containers of 2000 apple capacity, we found that the container assignment is the exact same as when the container size is 1000 when the maximum distance is 40 feet. Whereas only two containers are placed when the distance is set at 100 feet. We conclude that it is up to the farmers discretion to make the final tradeoff between far spaced and lesser number of containers, or tightly spaced containers that are easy to reach for the harvester based on our proposed assignments and visualization.

## 6. Conclusions

This work on yield estimation and visualization using deep learning develops an end-to-end framework that receives video footage of fruit trees and produces accurate yield estimates combined with GPS coordinates that are visually mapped. Using video footage, due to their efficiency, demands the addition of a tracking functionality in addition to object detection which is used in past works for yield estimation. In this work, we use YOLOv3 for object detection and the DeepSORT tracking algorithm for tracking. We modified DeepSORT to work more robustly on various fruits by implementing ResNet18 in place of the original proposed CNN. Our modification was successful and the framework performs well on various fruits from different views, producing high accuracy. While collecting apple data from the orchard, we recorded the GPS coordinates. We leverage this data to associate a set of frames with a respective coordinate, allowing us to visualize the yield information on a map. Using geospatial information, we were able to implement an efficient container placement algorithm that suggests optimal locations for containers in preparation for harvesting. Through the inclusion of both visualized yield estimates and optimal container placements, we are able to present farmers with a smart harvesting solution that aids them in understanding the states of their fields, and efficiently plan their logistics before harvest. We found that our work can be limited by the performance of our detector and some visual challenges such as object occlusion. We developed a strategy for fruit data annotation to tackle these challenges and diversify the detector training dataset for better performance. Although this strategy was successful and has significantly improved the detector performance, the detector remains the bottleneck in the proposed framework. Overall, the proposed framework is successful in producing accurate yield estimates and generating a mapping solution to improve the usability of fruit analysis to farmers, supporting decision making for better and efficient logistics management.

## Figures and Tables

**Table 1:** Comparison between different SSD models.

Method	Backbone	AP	FPS (Batch Size of 1)	GPU
YOLOv3-512 [19]	Darknet-53 [19,30]	42.4%	48.7	Telsa P100 (10 TFLOPS)
YOLOv3-608 [19]	Darknet-53 [19,30]	43%	43.1	Telsa P100 (10 TFLOPS)
EfficientDet-D3 [20]	EfficientNet-B3 [14]	47.2%	34.4	Tesla V100 (15.7 TFLOPS)
RetinaNet [21] (w/TRT)	SpineNet-49S-640 [13]	39.9%	85.4	Tesla V100 (∼30 TFLOPS)
RetinaNet [21] (w/TRT)	SpineNet-49-640 [13]	42.8%	65.3	Tesla V100 (∼30 TFLOPS)
CenterNet-HG [22]	Hourglass-104 [17]	45.1%	7.8	Titan XP (12 TFLOPS)
CenterNet-DLA [22]	DLA-34 [18]	41.6%	28	Titan XP (12 TFLOPS)

**Table 2:** The ResNet18 [31] architecture is used for DeepSORT’s feature extraction. The fully connected and Softmax layers are discarded.

Layer Name	Output Size	ResNet-18
conv1	112×112×64	7×7, 64, stride 2
conv2_x	56×56×64	$\frac{3\times 3\;\max\;\mathrm{pool}\;,\;\mathrm{stride}\;2}{\left[\begin{array}{c} 3\times 3,64\\ 3\times 3,64 \end{array}\right]\times 2}$
conv3_x	28×28×128	$\left[\begin{array}{c} 3\times 3,128\\ 3\times 3,128 \end{array}\right]\times 2$
conv4_x	14×14×256	$\left[\begin{array}{c} 3\times 3,256\\ 3\times 3,256 \end{array}\right]\times 2$
conv5_x	7×7×512	$\left[\begin{array}{c} 3\times 3,512\\ 3\times 3,512 \end{array}\right]\times 2$
average pool	1×1×512	7×7 average pool
fully connected	1000	512×1000 full connections
softmax	1000	

**Table 3:** A sample from the GPS data in an excel sheet after counting.

Lat	Lng	Count
43.91709	−78.6277	37
43.91709	−78.6277	61
43.91709	−78.6277	43
43.91709	−78.6277	43
43.91771	−78.6277	44
43.917711	−78.6277	66
43.917712	−78.6277	48
43.917714	−78.6277	55
43.917716	−78.6277	71

**Table 4:** Different cases that affect the appearance of an apple that we include in our training dataset.

Case	Image	Description
1	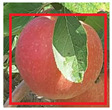	Apple is partly occluded by a leaf; we include the leaf in the image so the model can learn apples with some leaf features
2	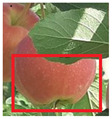	Half of the apple is occluded by a leaf; we include half the apple so the model can recognize what half an apple looks like and avoid over-training the model to start mistaking leaves for apples
3	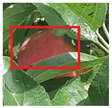	Apple is heavily occluded by leaves, we only include the visible part of the apple, improving the models ability to recognize partial apple features
4	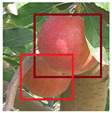	Apple is overlapping another apple; though this problem is normally mended as the video progresses and the angle changes, we still aim to ensure that the model is able to identify different apples detections that intersect with each other
5	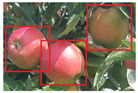	Apples under different lighting: we address the variance in natural lighting where some spots are bright, and others are very dim by including apples under different lighting conditions

**Table 5:** Results of the proposed pipeline running on a video clip of apples. We show the predicted count versus the actual count and compute the L1 Loss and Accuracy. The accuracy was low with the pretrained weights due to a significant overcount. Our correction mechanism substantially improved the accuracy, however fine tuning the weights led to the best performance.

Metrics	Pretrained	Pretrained and Corrected	Finetuned
Predicted/Ground Truth	523/342	299/342	313/342
L1 Loss	181	43	29
Accuracy	47.06%	87.43%	91.5%

**Table 6:** Results of the proposed pipeline running on a video clip of three neighboring rows of apples. We observed consistent performance across the three rows, with accuracy varying between 90–95%. This is consistent with the performance shown on the smaller scale apple detection in the earlier experiment.

Metrics	Apple Row 1	Apple Row 2	Apple Row 3
Predicted/Ground Truth	4375/4827	3647/3921	3530/3683
L1 Loss	452	276	153
Accuracy	90.6%	93.0%	95.8%

**Table 7:** Results of the proposed pipeline running on a video clip of pumpkins and oranges. The oranges are counted using pretrained YOLO weights and thus produce a lower accuracy of 79.3%. Since pumpkins are trained specifically on aerial views of pumpkins, including a sample from the experiment video, the accuracy was quite high.

Metrics	Pumpkin Counting	Orange Counting
Predicted/Ground Truth	219/233	96/121
L1 Loss 1	14	25
Accuracy	93.9%	79.3%

**Table 8:** All of the assigned containers are fully utilized. Note that while the last container has 77% utilization, this is because the remaining number of apples was 230 at that point, not 300, so 77% is the maximum utilization the container can reach.

Latitude	Longitude	Utilization of Container
43.91716	−78.62771	100%
43.91732	−78.62776	100%
43.9174	−78.62782	100%
43.91757	−78.62788	100%
43.91774	−78.62796	100%
43.91786	−78.628	100%
43.91805	−78.6281	100%
43.91818	−78.62814	100%
43.91833	−78.62821	100%
43.91853	−78.62829	100%
43.91865	−78.62834	100%
43.91872	−78.62838	77%

**Table 9:** None of the containers have 100% utilization due to the maximum distance restriction, however they’re still fairly highly utilized, thus no containers are wasted and the farmer may find this to be a favorable balance between even spacing of containers and properly utilizing the container capacities.

Latitude	Longitude	Utilization of Container
43.91743	−78.62783	97.4%
43.91785	−78.628	80%
43.91827	−78.62816	76.7%
43.91872	−78.62838	98.9%

**Table 10:** The containers are fully utilized, however the last container is only half full and is placed too close to the 3rd container, and the other three containers have high spacing between them. This is a less favorable option for the farmer as it adds extra time and effort for the harvesters.

Latitude	Longitude	Utilization of Container
43.91745	−78.62783	100%
43.91798	−78.62807	100%
43.91853	−78.62829	100%
43.91872	−78.62838	53%

## Data Availability

Not applicable.
